# Targeted Metabolomics Reveals Abnormal Hepatic Energy Metabolism by Depletion of β-Carotene Oxygenase 2 in Mice

**DOI:** 10.1038/s41598-017-15222-x

**Published:** 2017-11-07

**Authors:** Lei Wu, Xin Guo, Yi Lyu, Stephen L. Clarke, Edralin A. Lucas, Brenda J. Smith, Deana Hildebrand, Weiqun Wang, Denis M. Medeiros, Xinchun Shen, Dingbo Lin

**Affiliations:** 1 Department of Nutritional Sciences, Oklahoma State University, Stillwater, Oklahoma 74078 USA; 2 College of Food Science and Engineering, Nanjing University of Finance and Economics, Nanjing, 210046 China; 3 Department of Food, Nutrition, Dietetics, and Health, Kansas State University, Manhattan, KS 66506 USA; 4 Graduate School, University of Missouri, Kansas City, MO 64110 USA

## Abstract

β-carotene oxygenase 2 (BCO2) is a carotenoid cleavage enzyme located in the inner mitochondrial membrane. Ablation of BCO2 impairs mitochondrial function leading to oxidative stress. Herein, we performed a targeted metabolomics study using ultrahigh performance liquid chromatography-tandem mass spectroscopy and gas chromatography-mass spectroscopy to discriminate global metabolites profiles in liver samples from six-week-old male BCO2 systemic knockout (KO), heterozygous (Het), and wild type (WT) mice fed a chow diet. Principal components analysis revealed distinct differences in metabolites in the livers of KO mice, compared to WT and Het mice. However, no marked difference was found in the metabolites of the Het mouse liver compared to the WT. We then conducted random forest analysis to classify the potential biomarkers to further elucidate the different metabolomics profiles. We found that systemic ablation of BCO2 led to perturbations in mitochondrial function and metabolism in the TCA cycle, amino acids, carnitine, lipids, and bile acids. In conclusion, BCO2 is essential to macronutrient and mitochondrial metabolism in the livers of mice. The ablation of BCO2 causes dysfunctional mitochondria and altered energy metabolism, which further leads to systemic oxidative stress and inflammation. A single functional copy of BCO2 largely rescues the hepatic metabolic homeostasis in mice.

## Introduction

β-carotene oxygenase 2 (BCO2), a carotenoid cleavage enzyme, is conserved in primates and catalyzes asymmetrical cleavage of carotenoids at 9, 10 and 9′, 10′ double bond^1,2^. Interestingly, human genetic studies have revealed that mutations of *BCO2* are linked to alteration of circulating interleukin 18 (IL-18) level^4^, macular degeneration^5^, obesity^6^, and even cancer development^7^. The expression of BCO2 is significantly down-regulated in diabetic^8^ and obese mice^9^. Accordingly, intake of carotenoid-rich diet elevates the BCO2 expression and improves insulin sensitivity in obese C57BL/6 mice^8^. Further, the loss of BCO2 protein expression contributes to development of anemia in zebrafish, which is caused by apoptosis of erythrocyte precursors^10^. Still under debate is the enzymatic function of BCO2 in primates′ retina, due in part to lack of the correlation between carotenoid levels and BCO2 enzymatic activity in human retina^3^. Collectively, these studies indicate that BCO2 likely exerts other roles than solely as a carotenoid cleavage enzyme in mammals.

Energy homeostasis is maintained by a complex system, involving various signaling pathways and “nutrient sensors” in different types of tissues and/or organs. Any defect within these pathways or sensors can lead to metabolic disorders including type 2 diabetes, obesity and its associated metabolic syndromes. For instance, the 5′-adenosine monophosphate-activated protein kinase (AMPK) and mammalian/mechanistic target of rapamycin (mTOR) signaling play significant roles in the development of these metabolic disorders. AMPK, a whole-body energy sensor, modulates food intake and energy expenditure by responding to nutrient and hormonal signals. AMPK is activated when cellular energy is low (e.g. caloric restriction, exercise) and inhibited in a state of excess cellular nutrients (e.g. glucose, and fatty acids)^11,12^. What’s more, AMPK is down-regulated in obese and diabetic subjects^13,14^. mTOR, one of the downstream targets of AMPK, functions as an “intracellular nutrient sensor” which controls cell growth, protein synthesis and metabolism^15^. The activation of AMPK leads to inhibited mTOR signaling to suppress protein synthesis, which is an important regulatory mechanism by how AMPK maintains cellular energy during low energy condition^16^.

BCO2 is widely expressed in various types of tissues^17^ and is located in the inner mitochondrial membrane^18^. Recently, it has been demonstrated that ablation of BCO2 leads to mitochondrial dysfunction and subsequent cellular oxidative stress^19^. Our laboratory and others have also reported that intact BCO2 is essential for lipid metabolism in laboratory animals^9,10,17^. Moreover, it has been shown that whole body knockout (KO) of BCO2 leads to elevated food intake without increasing the body weight, which indicates that the energy homeostasis might be perturbed in KO mice^19^. However, the detail mechanism by which BCO2 status affects whole body energy metabolism still remains unknown. In this current study, the liver tissues of wild type (WT), BCO2 KO and heterozygous (Het) mice fed with a standard chow diet were subjected to targeted metabolomics and immunoblotting analysis. We sought to determine whether BCO2 protein was critical to energy metabolism in liver cells, and to investigate the effect of BCO2 status on mitochondrial function and subsequent energy metabolism.

## Results

### Global phenotypic changes in BCO2 KO mice

The daily food intake was measured from postnatal week 3 to week 6. The results showed food intake of Het mice was similar to KO mice, which was significantly higher than WT mice at 6 weeks of age (Data not shown)^19^. Interestingly, despite the higher food intake, the body weight of Het mice at 6-weeks-old was similar to WT mice, which was significantly lower than KO mice (Data not shown)^19^. Both KO and Het mice presented higher fasting blood glucose compared to age- and gender-matched WT mice (Table 1). However, the uric acid levels in KO and Het mice were significantly lower compared to WT mice (Table 1). Moreover, only KO mice presented a higher circulated non-esterified fatty acid (NEFA) level compared to WT and Het mice (Table 1). Whereas, no significant changes were found in triglyceride (TG) levels among the three groups (Table 1).

**Table 1:** Summary of phenotypic changes in BCO2 WT/KO/Het mice at 6-week-old fed with a chow diet.

Parameters	WT	KO	Het
Fasting blood glucose (mg/dL)	91.50 (7.63)^a^	118.71 (13.61)^b^	113.00 (19.34)^b^
NEFA (mEq/dL)	1.14 (0.12)^a^	1.39 (0.10)^b^	1.10 (0.10)^a^
TG (mg/dL)	70.60 (15.74)	77.80 (18.49)	79.00 (15.10)
Uric acid (mg/dL)	0.57 (0.15)^a^	0.14 (0.08)^b^	0.21 (0.04)^b^

Values are presented as means (SD), N = 6 mice/group, different letters indicate significant difference. NEFA, non-esterified fatty acids; TG, triglycerides; WT, wild type 129S6 mice; KO, β-carotene oxygenase 2 (BCO2) knockout; Het, BCO2 heterozygous mice.

### Targeted metabolomics profile of liver metabolites

**Figure 1:**
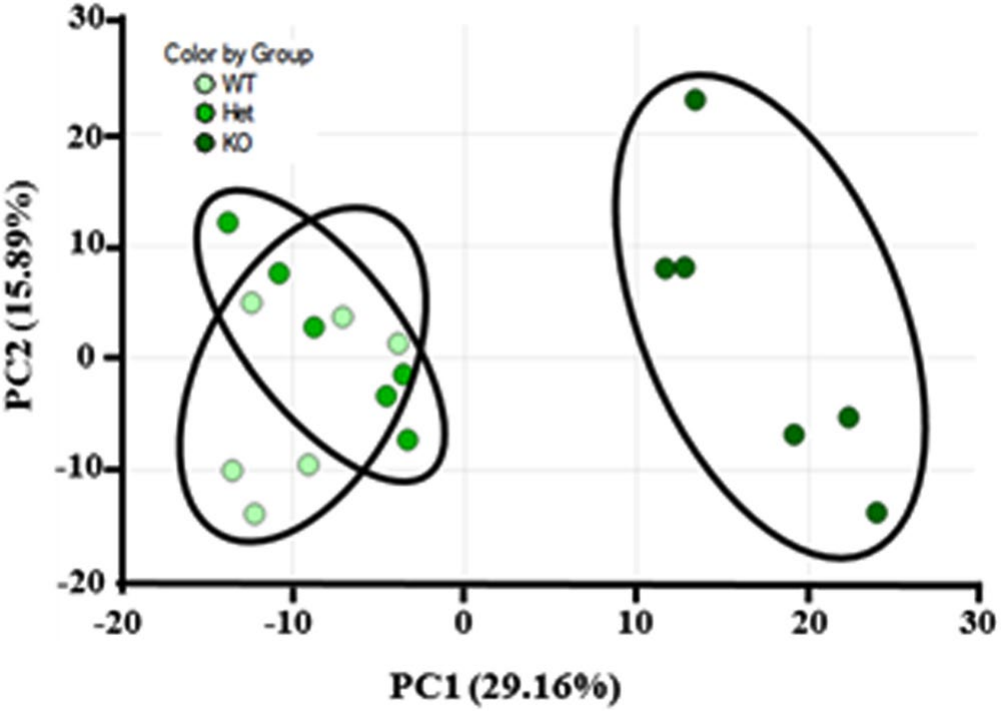
Principal component analysis (PCA) score blots of wild type (WT), β-carotene oxygenase 2 (BCO2) heterozygous (Het) and knockout (KO) liver metabolites data on 6-week-old. KO mice had very distinct PCA clustering in liver compared to WT and Het. However, the Het and WT mice showed similar PCA clustering (n = 6).

Liver targeted metabolomics by UPLC-MS/MS and GC-MS was used to investigate the distinct metabolic mechanism among KO, Het and WT mice at 6-week-old. Among the normalized intensities of metabolites extracted from liver samples, a total of 602 metabolites were identified by UPLC-MS/MS and/or GC-MS. Compared to WT and/or Het groups, 250 metabolites were found significantly different in BCO2 KO mice. There were only 99 metabolites significantly different between Het and WT groups (Supplemental Table 1). To discriminate the different metabolic profiles among KO, Het and WT groups, the principal components analysis (PCA) score plot was applied to visualize the sample distribution patterns. The two-dimensional scatter plot of the UPLC-MS/MS and GC-MS was defined by the first and second principle components PC1 (29.16%) and PC2 (15.89%). As shown in Fig. 1, the KO group was clearly separated from their age-matched WT and Het along PC1 and explained 45% of the total variation. Therefore, metabolic variability exists in the liver among different BCO2 genotypes of mice. However, livers from Het mice presenting one functional allele were not distinguishable from WT mice by PCA, suggesting that a single functional copy of BCO2 was possibly essential for maintaining metabolic homeostasis in mice.

### Markers of mitochondrial metabolism are altered in KO liver

**Figure 2:**
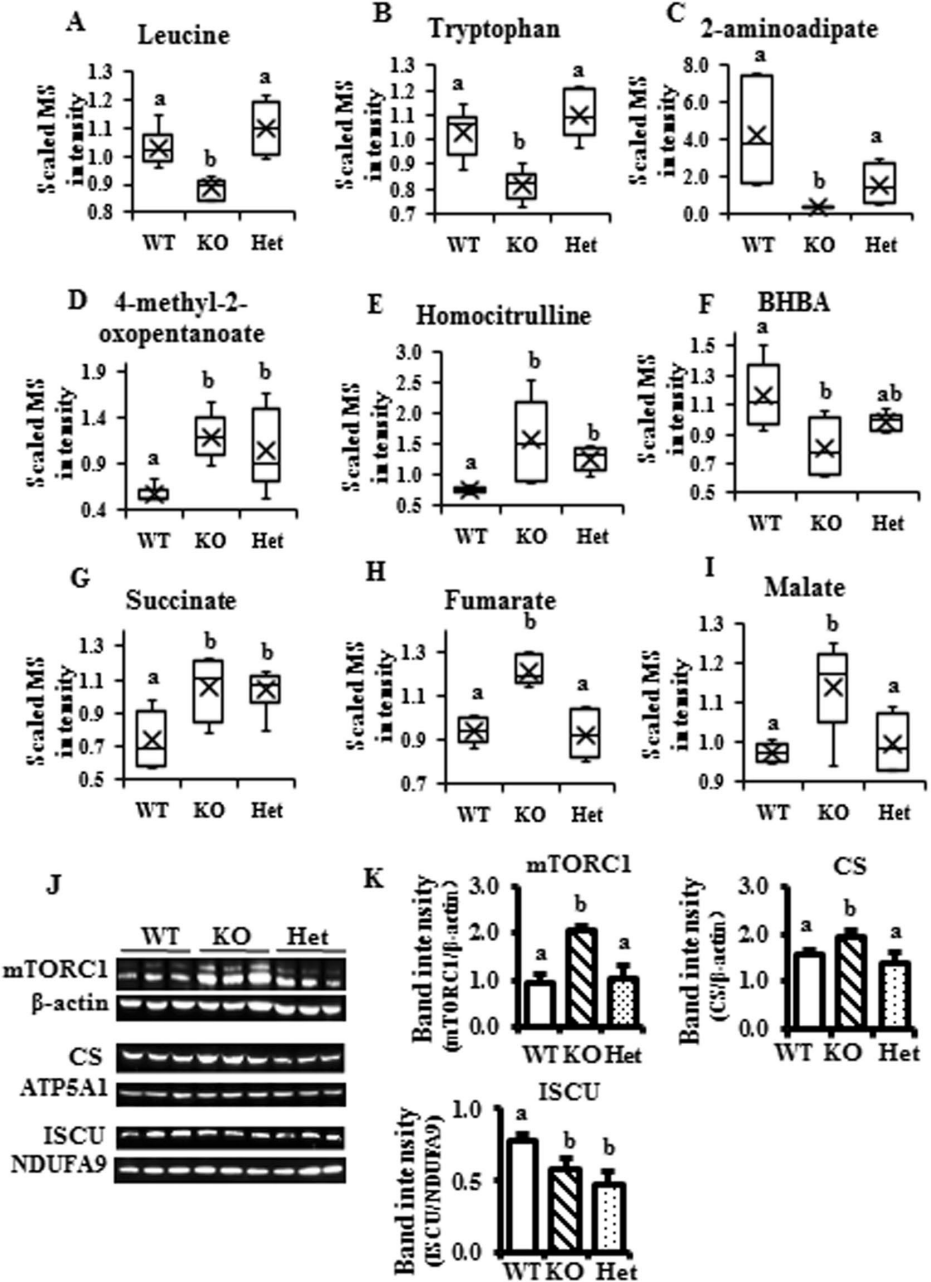
Altered mitochondrial metabolism markers in the β-carotene oxygenase 2 (BCO2) knockout (KO) mouse liver at 6 weeks of age. (**A–F**) Metabolites related to the amino acid oxidation. (**G–I**) Metabolites associated with the TCA cycle. **(J**) Expression of mitochondrial metabolism-related proteins by immunoblotting, and (**K**) quantification of listed proteins. Values are means ± SD. Data was analyzed by one-way ANOVA. n = 6. Different letters indicate significant difference between groups. P-value ≤ 0.05 is considered as significant. BHBA, β-hydroxybutyrate; mTORC1, mammalian/mechanistic target of rapamycin complex 1; CS, citrate synthase; ISCU, iron-sulfur cluster assembly enzyme; ATP5A1, ATP synthase, H^+^ transporting, mitochondrial F1 complex, alpha subunit 1; NDUFA9, NADH:ubiquinone oxidoreductase subunit A9; WT, wild type; Het, BCO2 heterozygous mice; KO, BCO2 knockout mice.

To gain insights into the metabolic mechanism involved in the significantly different metabolites in KO mice liver, the major metabolic pathways and possible physiological reactions of potential biomarkers were identified based on Human Metabolome Database (HMDB), KEGG database and related reference (See targeted metabolomics raw data in the supplemental Excel file and Fig. 2).

The results showed that several biomarkers of mitochondrial metabolism were significantly changed in KO mice liver based on targeted metabolomics results. The levels of leucine and tryptophan were significantly decreased in KO mice compared with WT and/or Het mice (Fig. 2A,B). Two-aminoadipate derived from lysine or tryptophan degradation, whose disposal are dependent on the mitochondrial function, were significantly decreased in KO compared to WT and Het liver samples (Fig. 2C). On the other hand, 4-methyl-2-oxopentanoate, the deamination product of leucine, was elevated in both KO and Het mice (Fig. 2D). Homocitrulline, formed when lysine is substituted for ornithine in the mitochondrial reaction catalyzed by ornithine transcarbamylase (OTC), was also significantly increased in KO and Het mice (Fig. 2E). These changes suggested that oxidation of amino acids, such as leucine, tryptophan and lysine, which are dependent on mitochondrial function, was restricted in KO mice, which also supports the significant decrease of ketone 3-hydroxybutyrate (BHBA) (Fig. 2F). Interestingly, metabolites associated with the TCA cycle, such as succinate, fumarate and malate, were significantly increased in the liver of KO but not WT or Het mice livers (Fig. 2G–I).

Immunoblotting analysis was further applied to confirm the findings from metabolomics analysis. The mammalian/mechanistic target of rapamycin 1 (mTOR1), an important biomarker for mitochondrial biogenesis, was significantly increased in KO mice livers compared to the livers of WT/Het mice (Fig. 2J,K). In consistent with the elevated levels of succinate, fumarate and malate, the protein level of citrate synthase (CS) was significantly increased KO mice, indicating the activated TCA cycle in KO mice compared to WT and Het mice (Fig. 2J,K). In contrast, the level of iron-sulfur cluster assembly enzyme (ISCU), which is important for mitochondrial respiration and energy production, such as mitochondrial respiratory complexes I, II, and III), was significantly decreased in both KO and Het mice, compared to the WT (Fig. 2J,K). In summary, while both metabolomics and immunoblotting analysis results demonstrated that BCO2 KO led to dysregulation of mitochondrial metabolism, one functional copy of BCO2 might be sufficient to maintain normal hepatic mitochondrial metabolism in mice.

### Indicators of oxidative stress and cysteine demand are elevated in KO liver

**Figure 3:**
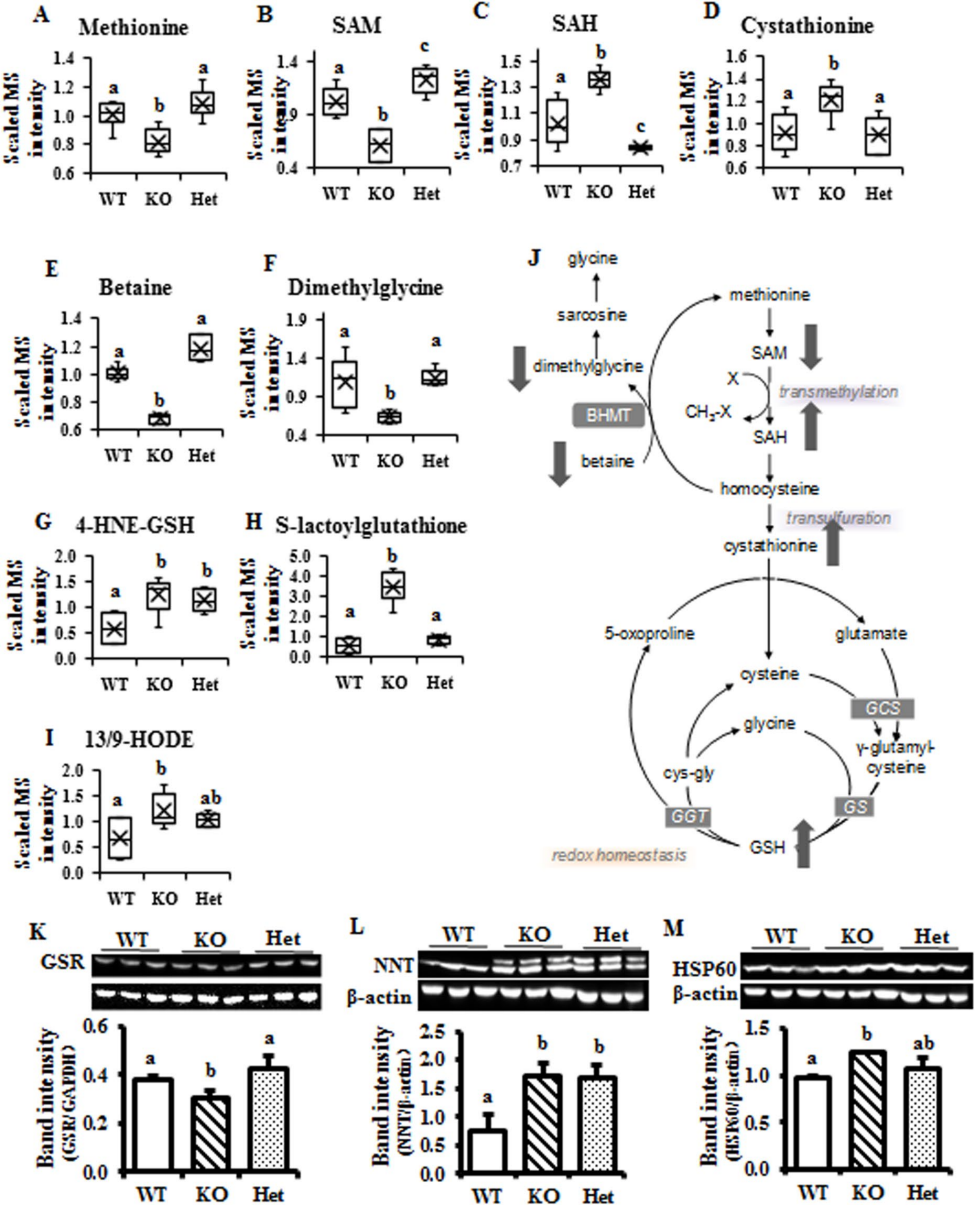
Oxidative stress markers are enhanced in the BCO2 KO mouse liver. (**A**–**F**) Metabolites associated with one carbon metabolism. (**G** and **H**) Glutathione-associated metabolites. (**I**) Metabolite associated with amino acid peroxidation. (**J**) Simplified scheme about one carbon and glutathione metabolism pathways. Protein expression of glutathione disulfide reductase (GSR) (**K**) nicotinamide nucleotide transhydrogenase (NNT) (**L**) and heat shock protein 60 (HSP60)(**M**) by immunoblotting and subsequent quantification. Values are means ± SD. Data was analyzed by one-way ANOVA. n = 6. Different letters indicate significant difference between groups. P-value ≤ 0.05 is considered as significant. SAM, S-adenosyl methionine; SAH, S-adenosylhomocysteine; 4-HNE-GSH, 4-hydroxy-2(E)-nonenal-glutathione; 13/9-HODE, 13/9-hydroxyoctadecadienoic acid; BHMT, betaine-homocysteine S-methyltransferase; GCS, γ-glutamylcysteine synthetase; GS, glutathione synthase; GGT, γ-glutamyltransferase; WT, wild type; Het, BCO2 heterozygous mice; KO, BCO2 knockout mice.

Compared to the WT and Het mice, hepatic levels of sulfur-containing amino acids, such as methionine, S-adenosylmethionine (SAM), S-adenosylhomocysteine (SAH), and cystathionine were significantly different in KO mice (Fig. 3A–D). In addition, betaine and dimethylglycine were significantly lower in KO mice livers compared to WT and Het mice, indicating the inhibited recycling of carbons between homocysteine and methionine (Fig. 3E,F).

The metabolism of sulfur-containing amino acids is important for one carbon transfer reactions catalyzed by methyltransferases as well as for converting methionine into cysteine and sulfhydryl-containing antioxidants such as glutathione. An elevated level of glutathione conjugates of reactive aldehydes, such as 4-hydroxy-nonenal-glutathione (4-HNE-GSH) and S-lactoylglutathione, indicated that homocysteine may have been predominantly converted to cysteine rather than back to methionine in BCO2 KO liver (Fig. 3G,H). Therefore, peroxidative stress was elevated in KO liver and more glutathione was needed to neutralize these reactive compounds. Thirteen-hydroxyoctaecadienoate (13-HODE or 9-HODE), which is a peroxidation product of linoleate, was also elevated in KO mice livers (Fig. 3I). This also confirmed promotion of oxidative stress in KO liver. A brief summary scheme is provided in Fig. 3J.

To further confirm the targeted metabolomics data, immunoblotting analysis on protein markers of oxidative stress were performed. The level of glutathione reductase (GSR), a mitochondrial protein functioning to maintain high levels of reduced glutathione in the cytosol, was significantly decreased in KO mice, however, no difference was found between WT and Het mice (Fig. 3K). NAD(P) transhydrogenase (NNT), which is considered as an oxidative stress marker, was significantly increased in KO/Het liver compared to WT (Fig. 3L). Heat shock protein 60 (HSP60), which is elevated under oxidative stress condition^20^, was clearly up-regulated in the liver tissues of KO and Het mice, compared to WT (Fig. 3M).

### The pentose phosphate pathway (PPP) is perturbed in KO mice

**Figure 4:**
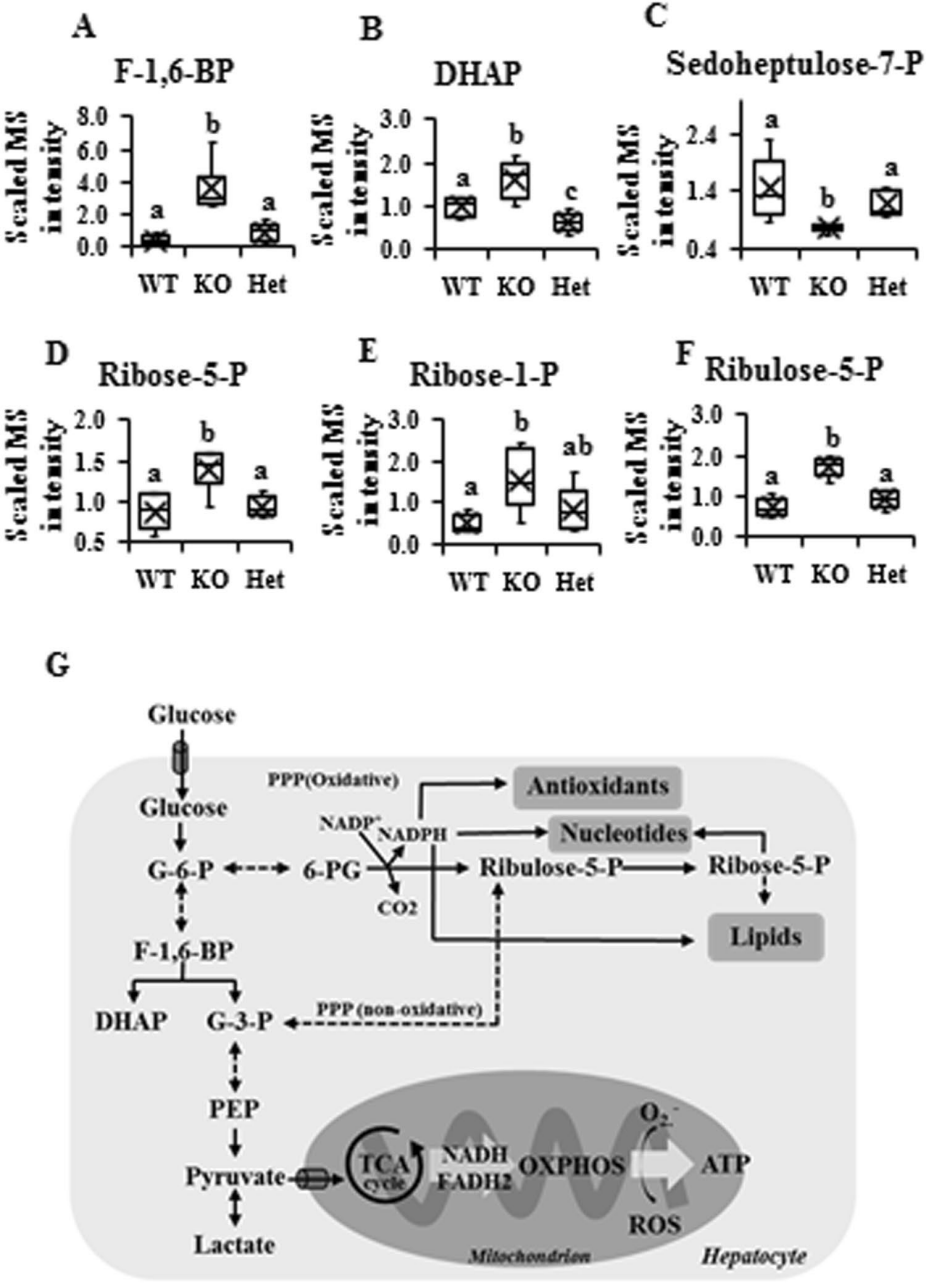
Pentose phosphate pathway (PPP) is altered in the β–carotene oxygenase 2 (BCO2) knockout (KO) liver. (**A**–**F**) Metabolites associated with PPP. (**G**) Simplified scheme about PPP in the liver. Values are means ± SD. Data was analyzed by one-way ANOVA. n = 6. Different letters indicate significant difference between groups. P-value ≤ 0.05 is considered as significant. F-1,6-BP, fructose-1,6-bisphosphate; DHAP, dihydroxyacetone phosphate; G-6-P, glucose 6-phosphate; 6-PG, 6-phosphogluconate; G-3-P, glyceraldehyde 3-phosphate; PEP, phosphoenolpyruvate; TCA cycle, the tricarboxylic acid cycle; OXPHOS, oxidative phosphorylation; WT, wild type; Het, BCO2 heterozygous mice; KO, BCO2 knockout mice.

Glycolysis and pentose phosphate pathway (PPP) are two major pathways for glucose metabolism. The targeted metabolomics data showed that the metabolites associated with glycolysis, such as fructose 1,6-bisphosphate (F-1,6-BP) and dihydroxyacetone phosphate (DHAP), increased significantly in KO compared to WT and/or Het mice, indicating more carbons fluxed into the TCA cycle (Fig. 4A,B).

The PPP provides cells with reducing equivalents such as NADPH, which preserves the intracellular levels of reduced glutathione (GSH) and provides substrate for lipid and nucleotide synthesis; with end product ribose-5-phosphate (ribose-5-P), the latter of which can also be used in the de novo synthesis of nucleotides^21,22^. In KO mice livers, the PPP metabolites, such as sedoheptupose-7-phosphate (sedoheptupose-7-P), ribose-5-phosphate (ribose-5-P), ribose-1-phosohate (ribose-1-P) and ribulose-5-P, were all elevated compared to WT and Het mice; however, no significant difference was observed between WT and Het mice livers (Fig. 4C–F). A brief summary scheme is provided in Fig. 4G.

### Carnitine and lipid metabolism are perturbed in BCO2 knockout mice

**Figure 5:**
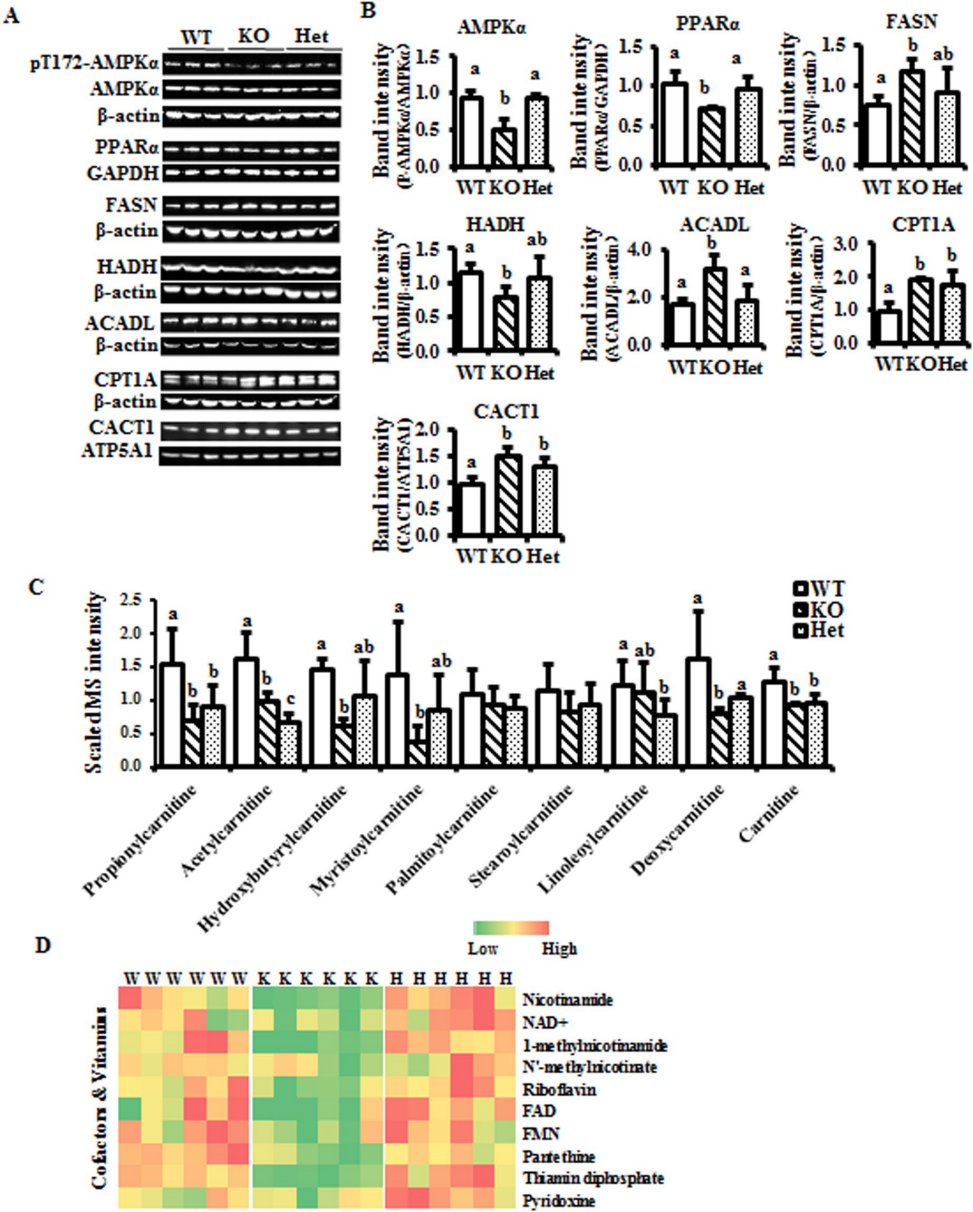
Energy metabolism is perturbed in the BCO2 KO liver. (**A**) Expression of proteins associated with energy metabolism, particularly lipid metabolism by immunoblotting. (**B**) Quantifications of listed proteins. (**C**) Metabolites associated with lipid metabolism. (**D**) Heat map of cofactors and vitamins associated with energy metabolism. Heat map only presents the significantly changes metabolites. Values are means ± SD. Data was analyzed by one-way ANOVA. n = 6. Different letters indicate significant difference between groups. P-value ≤ 0.05 is considered as significant. AMPKα, AMP activated protein kinase α; PPARα, peroxisome proliferator activated receptor α; FASN, fatty acid sunthase; HADH, mitochondrial hydroxyacyl-coenzyme A dehydrogenase; ACADL, long-chain specific acyl-CoA dehydrogenase; CPT1A, carnitine palmitoyltransferase 1α; CACT1, carnitine/acylcarnitine translocase 1; GAPDH, glyceraldehyde 3-phosphate dehydrogenase; ATP5A1, ATP synthase, H^+^ transporting, mitochondrial F1 complex, alpha subunit 1; W and WT, wild type; H and Het, BCO2 heterozygous mice; K and KO, BCO2 knockout mice.

AMPKα and PPARα, two key regulatory proteins in the energy metabolism pathway, were analyzed by immunoblotting. The activation of AMPKα, as indicated by phosphorylation of Thr172 in the AMPKα catalytic domain, was significantly decreased in KO mice. However, there was no significant difference found between WT and Het mice (Fig. 5A,B). Additionally, a similar pattern was found in the protein level of PPARα (Fig. 5A,B). The protein level of hydroxyacyl-coenzyme A dehydrogenase (HADH), which plays an essential role in the mitochondrial β-oxidation of short- or medium-chain fatty acids, was decreased in KO mice compared to the WT. However, Het mice could partially reverse the decreased level of HADH compared to WT mice (Fig. 5A,B). In contrast, the protein level of long-chain specific acyl-CoA dehydrogenase (ACADL), which involves in the mitochondrial β-oxidation of long chain fatty acids, was markedly elevated in KO compared to WT and Het mice (Fig. 5A,B). Moreover, the protein levels of carnitine palmitoyltransferase I (CPT1A) and carnitine/acylcarnitine translocase (CACT1), the key enzymes in the fatty acid β–oxidation pathway for long-chain fatty acids transport across the mitochondrial inner membrane and into mitochondria in exchange for free carnitine, were significantly increased in the KO and Het compared to WT mice (Fig. 5A,B). For lipid synthesis, fatty acid synthase (FASN) was increased significantly in the KO mouse liver, compared to the WT, but not the Het. The hepatic FASN level was similar in the Het and WT mice (Fig. 5A,B).

The targeted metabolomics data were further subjected to energy-associated pathway analysis. The results revealed that carnitine metabolism was downregulated in both BCO2 KO and Het compared to WT mice (Fig. 5C). In regard to carnitine-associated metabolites, carnitine, acyl-carnitines (e.g., propionylcarnitine, hydroxybutyrylcarnitine, myristoylcarnitine, deoxycarnitine) and acetylcarnitine were all decreased in both KO and Het compared to WT mice (Fig. 5C). Consistently, vitamins relevant to energy metabolism, were found to be downregulated in KO mice. However, no significant difference was found between WT and Het mice (Fig. 5D).

### Sterol metabolism is perturbed in the BCO2 KO liver

**Figure 6:**
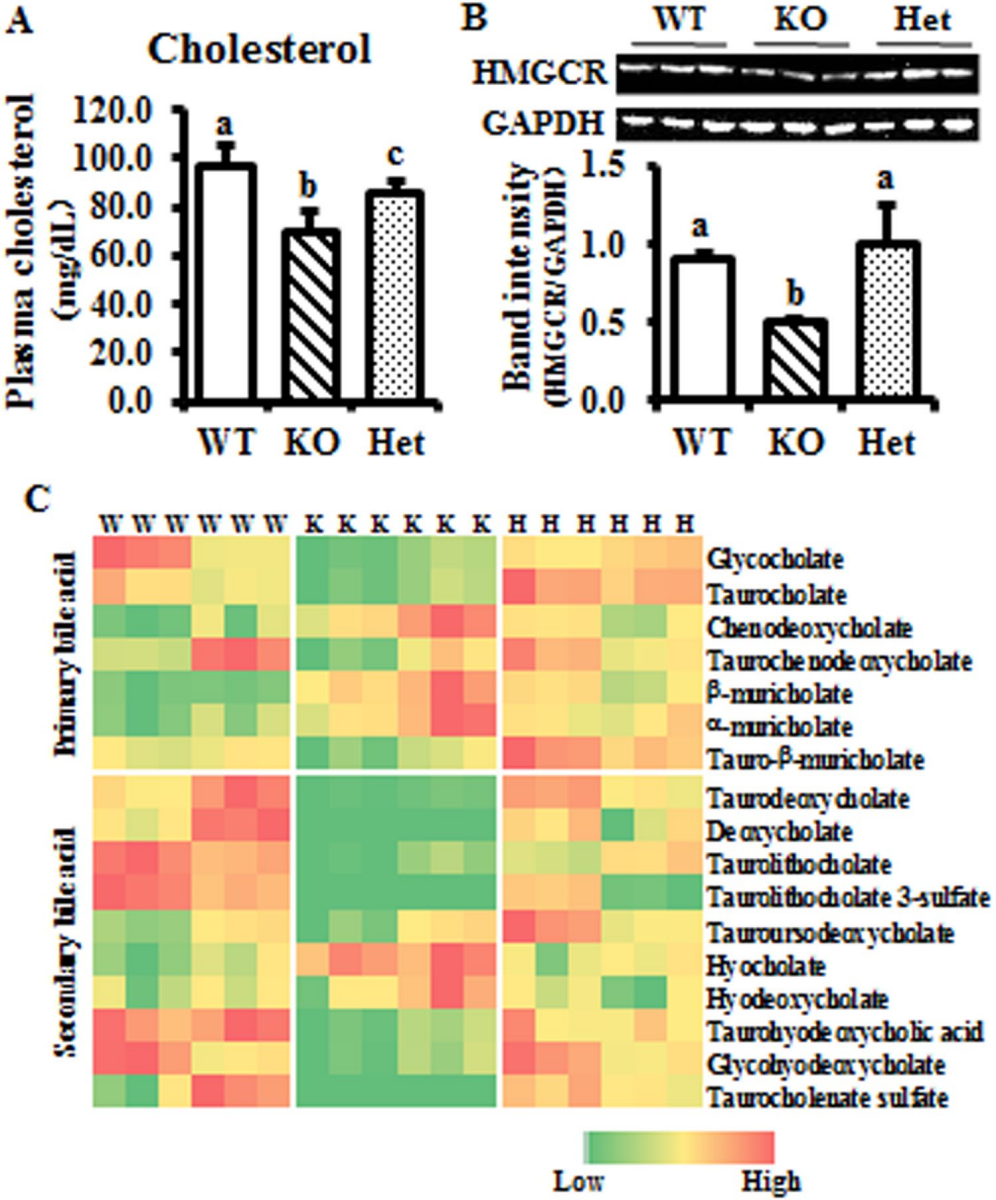
Cholesterol and bile acid metabolism is perturbed in KO liver. (**A**), plasma cholesterol level is low in KO mice. (**B**), expression of HMGCR is decreased in KO liver. (**C**), significantly changes metabolites associated with sterol and bile acid metabolism. Heat map only presents the significantly changes metabolites. Values are means ± SD. Data was analyzed by one-way ANOVA. n = 6. Different letters indicate significant difference between groups. P-value ≤ 0.05 is considered as significant. GAPDH, glyceraldehyde 3-phosphate dehydrogenase; HMGCR, 3-hydroxy-3-methylglutaryl-coenzyme A reductase; W and WT, wild type; H and Het, BCO2 heterozygous mice; K and KO, BCO2 knockout mice.

Different from the elevation of NEFA and glucose levels, the plasma cholesterol level was decreased in KO and Het compared to WT mice. However, the Het mice exhibited higher cholesterol level compared to KO mice (Fig. 6A). The protein level of 3-hydroxy-3-methylglutaryl-coenzyme A reductase (HMGCR), which catalyzes the rate-limiting step of cholesterol synthesis, (i.e., conversion of 3-hydroxy-3-methylglutaryl-coenzyme A (HMG-CoA) to mevalonate), was decreased in KO compared to WT and Het mice (Fig. 6B).

Bile acid synthesis via the ‘alternative’ acidic pathway involving 7-alpha-hydroxy-3-oxo-4-cholestenoate (7-HOCA) may have been more active in KO mice livers as the level of chenodeoxycholate was significantly elevated^23^ (Fig. 6C). Other primary bile acids, such as taurine-conjugate taurocholate, were reduced in KO mice livers, and, secondary bile acids, such as deoxycholate, tended to be significantly decreased in livers from KO mice (Fig. 6C). Data suggested that systemic depletion of BCO2 perturbed sterol metabolism in KO mice.

## Discussion

Metabolic disorder, which is considered to be a complex disease involving the systemic dysregulation of mitochondrial function, energy metabolism, signaling transduction, etc., is one of the major factors that contribute to the development of modern chronic diseases, such as type 2 diabetes, obesity and even cancer^24–26^. Therefore, identifying metabolic biomarkers can contribute to improved prognostication, diagnostics and therapy. BCO2, an important carotenoid cleavage enzyme, resides in the inner mitochondrial membrane^18^. However, it is shown that ablation of BCO2 did not affect hepatic vitamin A metabolism in mice at 6 weeks of age^19,27^. The hepatic retinol level in BCO2 KO mice is the same as that in the WT. And hepatic retinoid acid and retinyl esters levels are not altered either (unpublished data). Therefore, despite no apparent changes in pro-vitamin A carotenoid metabolism observed in BCO2 KO mice, the other roles of BCO2 in directly regulation of mitochondrial function and energy metabolism need to be more elucidated. Targeted metabolomics provide a novel and sensitive approach to study the disturbed metabolic pathways and biomarkers. Here, we employed targeted metabolomics to profile metabolites in the livers of WT, KO, Het mice at 6-week-old fed with a standard chow diet. The aims of this study were as follows: 1) to identify metabolic characteristics that could distinguish the KO, Het and WT mice; and, 2) to identify the possible metabolic pathways that could explain specific phenotype of KO mice. The results in this current study showed clear distinct metabolic patterns between BCO2 KO, Het and WT mice, although there is considerable overlap between WT and Het mice.

Random forest analysis showed broad signs of disrupted or altered mitochondrial function in the livers of KO mice, which is consistent with previous findings^10,19^. The present results indicated that oxidation of amino acids, such as leucine, tryptophan, and lysine, was restricted in KO mice livers - a hypothesis perhaps supported by the significant decrease of the BHBA. BHBA can also be derived from acetyl-CoA derived from fatty acid β-oxidation^28^, which was also shown to be down-regulated in the KO mice livers (Fig. 5). However, metabolites associated with the TCA cycle, such as succinate, fumarate, and malate, were increased in KO compared to WT mice liver indicating that either more carbons were introduced into the TCA cycle in KO relative to WT mice livers. Previous studies have shown that modulation of BCO2 activity and/or its protein expression leads to mitochondrial dysfunction^19^, so the changes in mitochondrial oxidative metabolites could be modest indications of mitochondrial stress in this animal model^8,29^.

Mitochondrial dysfunction links to oxidative stress and metabolic disorders^30,31^. Markers of oxidative stress, such as the lipid peroxidation marker 4-HNE-GSH^32^, protein markers NNT^33^ and HSP60^34^, were all elevated in KO mice livers, which is consistent with earlier findings that lack of BCO2 leads to oxidative stress^10,19^. The metabolic changes, such as decreased SAM/betaine/dimethylglycine, and elevated SAH/cystathionine, suggested that homocysteine may have been predominantly converted to cysteine rather than back to methionine. This was further confirmed by elevated level of glutathione conjugates of reactive aldehydes, such as S-lactoylglutathione and 4-HNE-GS. Cells, when physiologically exposed to a high oxidative burden, must have a high oxidative PPP flux to prevent constant NADPH usage^22^. Intriguingly, the activated oxidative PPP indicated the oxidative stress condition in livers of KO mice.

BCO2 KO mice presented higher food intake, however, the body weight gain was not higher than WT mice^19^, indicating the disturbed energy metabolism^35^. Moreover, the fasting blood glucose and circulating NEFA levels were significantly higher in the KO and Het mice compared to WT mice, although no significant difference was found in the NEFA level between WT and Het mice. Collectively, these results indicate that ablation of BCO2 leads to metabolic disorder, which might be caused by dysregulated mitochondrial function. Previous studies demonstrated that mitochondrial dysfunction leads to elevated ROS production^29,36^, which further impairs insulin signaling to inhibit activation of AMPKα^37^. Intracellular energy levels is sensed by AMPKα activation^38^. When cells are over-nourished (e.g., overnutrition), the increase in ATP levels inhibits AMPKα activity. The inhibition of AMPKα activity functions to activate mTOR complexes^39^. Following activation, mTOR complexes promote fatty acid synthesis and shut down catabolic processes such as fatty acid β-oxidation^40^.

The increase in fatty acid synthesis can also be supported by the activated oxidative PPP, as NADPH is a substrate for lipid synthesis. Carnitine and acylcarnitines, (e.g., propionylcarnitine, acetylcarnitine, etc.), were present at lower concentrations in the livers of KO and Het mice compared to age- and gender-matched WT mice (Fig. 5C). Carnitines, together with the transporters CPT1 and CACT1, play key roles in transporting long-chain fatty acids into the mitochondrial matrix, and further involve in β-oxidation and energy metabolism. In this study, the metabolites associated with carnitine biosynthesis and metabolism were all down-regulated in KO mice. Consistently, the levels of energy-metabolizing vitamins and the metabolites were also decreased in the KO mice. The level of PPARα, a major regulator of lipid metabolism in the liver^41^, was decreased in KO mice, indicating the inhibition of fatty acid utilization. However, the transporters CPT1A and CACT1, and ACADL, the key enzyme for long-chain fatty acid β-oxidation, were up-regulated to compensate for the lower levels of carnitines and acylcarnitines.

Similar to lipid metabolism, sterol metabolism appeared to be perturbed in KO mice. The plasma level of cholesterol was significant lower in KO compared to WT and Het mice. Additional markers associated with sterol metabolism and the conversion of cholesterol to primary and secondary bile acids were all down-regulated in the liver samples of KO mice. For starters, the increase of HMG (data not shown) and suppressed expression of HMGCR protein suggested that the rate-limiting step of cholesterol synthesis (i.e., the conversion of HMG-CoA to mevalonate by HMGCR^42^) was restricted in KO liver leading to the alternative disposal of HMG-CoA via formation of HMG. In this study, the protein metabolism was also altered in KO mice, as shown by significantly lower levels of dipeptides in liver (Supplemental Fig. 2) and uric acid in plasma.

We also combined metabolomics with immunoblotting biochemical analysis to dissect the metabolism pathway changes in WT, KO and Het mice. The resulting data demonstrated differences among those three strains. Broad changes in metabolites associated with mitochondrial metabolism and oxidative pathways were noted in the livers of KO mice. Differences in metabolites associated with PPP, carnitine and lipid metabolism were also presented in KO mice liver. Signs pointed to a range of changes in sterol synthesis, the conversion of cholesterol into primary bile acids, and the transformation of primary bile acids to their secondary forms. Largely, a single copy of the BCO2 could be important for hepatic metabolome homeostasis.

In summary, while there is evidence that the metabolism of macronutrients, including glucose, lipid, sterol, and protein (Supplemental Fig. 2), is disturbed in the KO mice compared to WT and Het mice. There is need for future studies to investigate the associated metabolic pathways specific to protein metabolism. In addition, further studies will be conducted to validate the relationship between sterol synthesis biomarkers and BCO2 in both obese and diabetic mice, as BCO2 level was significantly lower in these mouse subjects. Therefore, future studies are warranted to determine whether BCO2 represents a potential therapeutic target in maintaining mitochondrial homeostasis and energy metabolism that would be beneficial to prevent the risk of the development of relevant metabolic disorders in humans.

## Methods

### Animal and animal care

Whole body BCO2 knockout and heterozygous in 129S6 background mice (KO, Het) and 129S6 mice (WT) were husbanded in Oklahoma State University (OSU, Stillwater, OK, USA) Laboratory Animal Research facility. All the mice were housed under a daily 12-hour light/dark cycle, regulated temperature (21 ± 2 °C) and humidity (50 ± 5%). Mice, fed a normal chow diet (AIN 93 M) from Research Diets, at 6-week-old were used for current study. All animal protocols were approved by the Institutional Animal Care and Use Committee (IACUC) at Oklahoma State University. All methods were performed in accordance with the relevant guidelines and regulations by the Association for Assessment and Accreditation of Laboratory Animal Care International (AAALAC), the United States Department of Agriculture (USDA) and the National Institutes of Health (NIH), USA. Blood glucose was measured through the tail vein as described elsewhere^34^. Subsequently, the mice were euthanized to collect plasma and liver samples, which were stored under −80 °C for future analysis.

### Plasma parameters measurements

Plasma concentrations of total cholesterol, uric acid, TG and NEFA were measured using a BioLis 24i automated analyzer (Carolina Chemistry, NC, USA).

### Liver sample preparation for targeted metabolomics

Liver samples were collected from 6-week-old male mice from each group after 6-hour fasting. Liver samples were carefully dissected to avoid contamination of surrounding tissues. After washing with cold sterile PBS, 100 mg of liver samples were frozen in liquid nitrogen and delivered in dry ice to Metabolon, Inc (Durham, NC) for targeted metabolomics. The remaining liver samples were saved under −80 °C for future analysis.

For targeted metabolomics, samples were prepared using the automated MicroLab STAR® system from Hamilton Company. The resulting extract was divided into four portions: one for analysis by ultrahigh performance liquid chromatography-tandem mass spectroscopy (UPLC-MS/MS) with negative ion mode electrospray ionization, one for analysis by UPLC-MS/MS with positive ion mode electrospray ionization, one for analysis by gas chromatography-mass spectroscopy (GC-MS) and the last one for backup. For liquid chromatography analysis, the samples were stored overnight under nitrogen before analyzing. For gas chromatography analysis, the samples were dried under vacuum overnight before analyzing. The experimental design and data processing workflow is illustrated in Supplemental Fig. 1.

### UPLC-MS/MS and GC-MS conditions

For liquid chromatography, a Waters ACQUITY ultra-performance liquid chromatography (UPLC) coupled with a Thermo Scientific Q-Exactive high resolution/accurate mass spectrometer was used to analyze liver samples. Chromatographic separation was achieved using dedicated columns (Waters UPLC BEH C18–2.1 × 100 mm, 1.7 µm). One aliquot was analyzed using basic negative ion optimized conditions and acidic positive ion optimized conditions in two separate injections using different dedicated columns (Waters UPLC BEH C18–2.1 × 100 mm, 1.7 µm). Extracts reconstituted in acidic conditions were gradient eluted from a C18 column using water and methanol containing 0.1% formic acid. The basic extracts were similarly eluted from C18 using water and methanol with 6.5 mM ammonium bicarbonate. The third aliquot was analyzed via negative ionization following elution from a HILIC column (Waters UPLC BEH Amide 2.1 × 150 mm, 1.7 µm) using a gradient consisting of acetonitrile and water with 10 mM ammonium formate solution. The scan range was from 80–1000 *m/z* for UPLC-MS/MS.

For gas chromatography, pretreated samples were separated on a 5% diphenyl / 95% dimethyl polysiloxane fused silica column (20 m × 0.18 mm ID; 0.18 um film thickness) with a temperature ramp from 60° to 340 °C in a 17.5 min period and helium as carrier gas. Samples were analyzed on a Thermo-Finnigan Trace DSQ fast-scanning single-quadrupole mass spectrometer using electron impact ionization (EI) and operated at unit mass resolving power. The scan range was from 50–750 *m/z* for GC-MS.

### Targeted metabolomics data processing

Raw data was extracted, peak-identified and QC processed by Metabolon, Inc (Durham, NC). Compounds were identified by comparison to library entries of purified standards or recurrent unknown entities. In this present study, a total of 602 compounds of known identity (named metabolites) in liver were detected and listed in Supplemental Excel Sheet 1. Following log transformation and imputation of missing values, was used to identify metabolites that differed significantly between experimental groups. A summary of the numbers of metabolites that reached statistical significance (p ≤ 0.05), as well as those approaching significance (0.05 < p < 0.10), is shown in Supplemental Table 1.

### Immunoblotting analysis

For immunoblotting analysis, liver samples were homogenized in a cell lysis buffer containing 20 mM Tris, pH 7.5, 0.5 mM EDTA, 0.5 mM EGTA, 0.5% Triton X-100 and 1% protease/phosphatase inhibitor. The homogenates were sonicated and centrifuged under 8,000 × g, 4 °C, 10 minutes. The supernatants were saved under −80 °C for future immunoblot analysis. Bicinchoninic acid assay (BCA assay, Pierce, Rockford, IL) was used to measure the protein concentration. Equal amounts of liver protein samples were separated by sodium dodecyl sulfate polyacrylamide gels (SDS-PAGE) and then followed by immunoblotting as described previously^8,9^. To maximize the utilization of the immunoblotting membranes, the blots at least were cut into two parts according to the molecular weights of the target proteins and reused for a couples of times after being stripped with Thermo Scientific Restore PLUS Western Blot Stripping Buffer (Thermo Scientific Pierce, Rockford, IL, USA). Immunoreactive bands were detected by chemiluminescence (ECL, Thermo Scientific Pierce, Rockford, IL, USA) and visualized by the FluorChem R Imager System (ProteinSimple, San Jose, CA, USA). Total pixel intensity of each protein band was normalized to a loading control for graphing and statistical analysis. Protein markers associated with different metabolic pathways were tested. Antibodies against mammalian/mechanistic target of rapamycin complex 1 (mTORC1), citrate synthase (CS), carnitine/acylcarnitine translocase 1 (CACT1), ATP synthase, H^+^ transporting, mitochondrial F1 complex, alpha subunit 1(ATP5A1); NADH:ubiquinone oxidoreductase subunit A9 (NDUFA9); iron-sulfur cluster assembly enzyme (ISCU), nicotinamide nucleotide transhydrogenase (NNT), 60 kDa heat shock protein (HSP60), hydroxyacyl-coenzyme A dehydrogenase (HADH), long-chain specific acyl-CoA dehydrogenase (ACADL), carnitine palmitoyltransferase I (CPT1A), glyceraldehyde-3-phosphate dehydrogenase (GAPDH) and β-actin were purchased from ProteinTech Group (Rosemont, IL, USA). Antibodies against glutathione disulfide reductase (GSR) and peroxisome proliferator-activated receptor alpha (PPARα) were purchased from Santa Cruz Biotechnology (Dallas, TX, USA). Antibodies against AMP-activated protein kinase alpha [p-AMPKα(T172)/AMPKα], fatty acid synthase (FASN) and 3-hydroxy-3-methylglutaryl-coenzyme A reductase (HMGCR) were purchased from Cell Signaling Technology (Danvers, MA, USA). β-actin and glyceraldehyde 3-phosphate dehydrogenase (GAPDH), ATP5A1, and NDUFA9 were applied as loading controls in this present study.

### Statistical analysis

For metabolomics-based studies, two types of statistical analysis were performed, including significance tests and classification analysis. Standard statistical analyses were performed in ArrayStudio on log transformed data. Otherwise, the JMP or R were used for those analyses not fitted in ArrayStudio. Two-sided Welch’s two-sample t-test was used in present study to test whether two means are different from two independent populations. In metabolomics-based studies, p values were adjusted with the use of false discovery rate (q-value) to consider the multiple comparisons that normally occur. Principal components analysis (PCA) is an unsupervised analysis that reduces the dimension of the data to show whether or not a differentiation or grouping exists between different sample groups^43^. Random forest is a supervised classification technique^44^. To determine which metabolites make the largest contribution to the classification, the “Mean Decrease Accuracy” (MDA) “ was computed^45^. The top 30 metabolites with larger MDA values in the list were potentially worthy of further investigation and listed in Supplemental Excel Sheet 2. For the other analysis, one-way ANOVA was used to test whether at least two means are equal or whether at least one pair of means is different. Differences were considered as significant when p value ≤ 0.05.

### Data Availability

All data generated or analyzed during this study are included in this published article (and its Supplementary Information files). Raw datasets and protocols generated are available from the corresponding author on reasonable request.

## Electronic supplementary material

Supplemental figures

Dataset 2
