# Supplementary material for: Targeted Metabolomics Reveals Abnormal Hepatic Energy Metabolism by Depletion of β-Carotene Oxygenase 2 in Mice
Source: Sci Rep. 2017 Nov 7;7:14624. doi: 10.1038/s41598-017-15222-x (PMC5677115; doi:10.1038/s41598-017-15222-x)
Supplement: Supplementary file 1 — Supplemental figures [file 41598_2017_15222_MOESM1_ESM.pdf]

Supplemental Figures

Targeted Metabolomics Reveals Abnormal Hepatic Energy Metabolism by Depletion of β-Carotene Oxygenase 2 in Mice

Lei Wu, Xin Guo, Yi Lyu, Stephen Clarke, Edralin Lucas, Brenda J Smith, Deana Hildebrand, Weiqun Wang, Denis Medeiros, Xinchun Shen, Dingbo Lin

Department of Nutritional Sciences, Oklahoma State University, Stillwater, Oklahoma 74078, USA

Supplemental Table 1 Summary of the numbers of metabolites identified with significant changes in the livers of wild type (WT), β-carotene oxygenase 2 (BCO2) heterozygous, and/or BCO2 knockout (KO) mice

Statistical Comparisons			
Welch's Two-Sample *t*-Test	KO / WT	KO / Het	Het / WT
Total Metabolites ($p \leq 0.05$, ↑↓)	250 (**108** | **142**)	250 (**103** | **147**)	99 (**65** | **34**)
Total Metabolites ($0.05 < p < 0.10$, ↑↓)	36 (**18** | **18**)	42 (**10** | **32**)	45 (**30** | **15**)

The numbers in red show metabolites with increased contents, while the numbers in green indicate metabolites with decreased contents

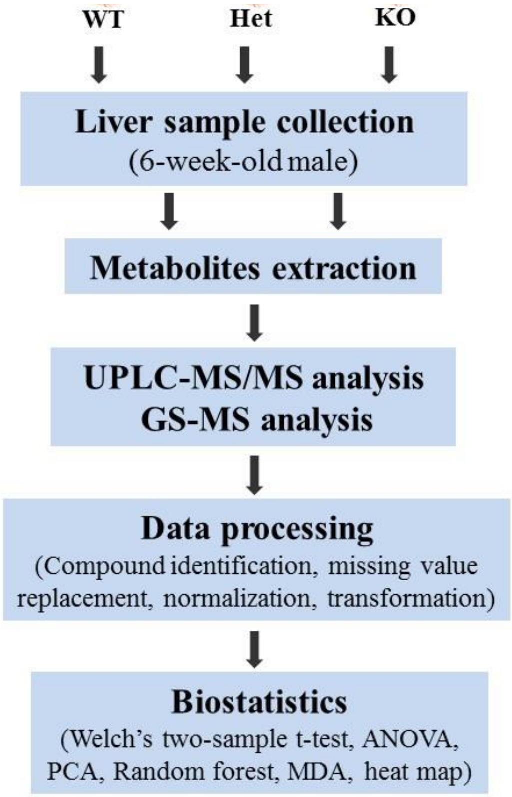

Supplemental Figure 1. Summary of the experimental design. Liver metabolites from 6-week-old male wild type (WT), β-carotene oxygenase 2 (BCO2) heterozygous (Het) and BCO2 knockout (KO) mice were subjected to UPLC-MS/MS and GS-MS analysis followed by data processing and biostatistics analysis (n = 6).

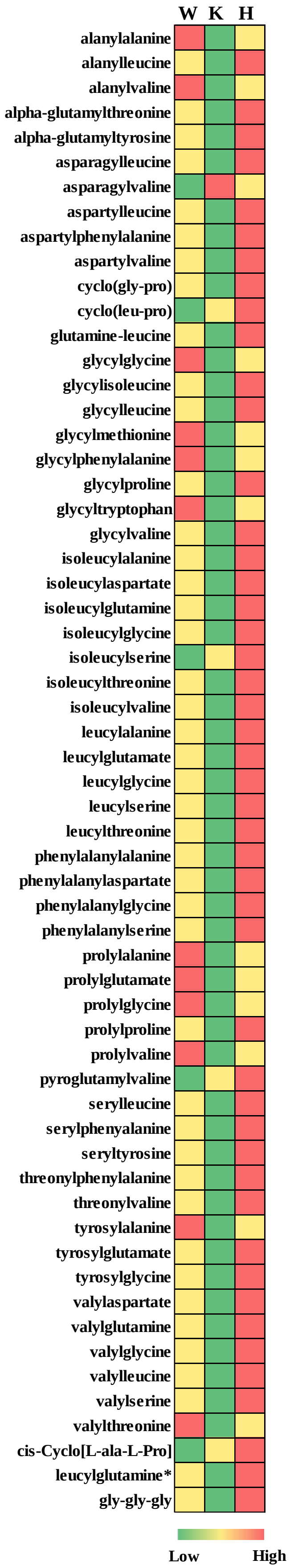

Supplemental Figure 2. Heat map of metabolites associated with peptides metabolism. Liver metabolites from 6-week-old male WT, Het and KO mice were subjected to UPLC-MS/MS and GS-MS analysis followed by data processing and biostatistics analysis (n = 6). W, wild type; H, β-carotene oxygenase 2 (BCO2) heterozygous mice; K, BCO2 knockout mice
